# Therapeutic Characterization and Efficacy of Bacteriophage Cocktails Infecting *Escherichia coli*, *Klebsiella pneumoniae*, and *Enterobacter* Species

**DOI:** 10.3389/fmicb.2019.00574

**Published:** 2019-03-21

**Authors:** Prasanth Manohar, Ashok J. Tamhankar, Cecilia Stalsby Lundborg, Ramesh Nachimuthu

**Affiliations:** ^1^Antibiotic Resistance and Phage Therapy Laboratory, Department of Biomedical Sciences, School of Bioscience and Technology, Vellore Institute of Technology, Vellore, India; ^2^Global Health-Health Systems and Policy: Medicines, Focusing Antibiotics, Department of Public Health Sciences, Karolinska Institutet, Stockholm, Sweden; ^3^Indian Initiative for Management of Antibiotic Resistance, Mumbai, India

**Keywords:** bacteriophage genome, *E. coli*, *Klebsiella pneumoniae*, *Enterobacter cloacae*, phage cocktail, phage therapy

## Abstract

Infections due to antibiotic resistant bacteria are increasing globally and this needs immediate attention. Bacteriophages are considered an effective alternative for the treatment of bacterial infections. The aim of this study was to isolate and characterize the bacteriophages that infect *Escherichia coli*, *Klebsiella pneumoniae*, and *Enterobacter* species. For this, clinical bacterial isolates of the mentioned species were obtained from diagnostic centers located in Chennai, Tamil Nadu, India. The bacteriophages were isolated from sewage water samples collected from Tamil Nadu, India. Phage isolation was performed using enrichment method and agar overlay method was used to confirm the presence of bacteriophages. All the phages were characterized for their life cycle parameters, genome analysis, and *in vitro* phage cocktail activity. The three bacteriophages exhibited broad host range activity: *Escherichia* virus myPSH2311 infecting *E. coli* belonging to six different pathotypes, *Klebsiella* virus myPSH1235 infecting *K. pneumoniae* belonging to four different serotypes and *Enterobacter* virus myPSH1140 infecting four different species of *Enterobacter*. Morphological observations suggested that the bacteriophages belonged to, *Phieco32virus* (*Escherichia* virus myPSH2311), *Podoviridae* (*Klebsiella* virus myPSH1235), and *Myoviridae* (*Enterobacter* virus myPSH1140). The life cycles (adsorption, latent period, and cell burst) of *Escherichia* virus myPSH2311, *Klebsiella* virus myPSH1235 and *Enterobacter* virus myPSH1140 were found to be 26, 40, and 11 min, respectively. Genomic analysis revealed that *Escherichia* virus myPSH2311 is closely related to *Escherichia* phage vB_EcoP_SU10, *Klebsiella* virus myPSH1235 is closely related to *Klebsiella* phage vB_KpnP_KpV48 and *Enterobacter* virus myPSH1140 is closely related to *Enterobacter* phage PG7 and *Enterobacter* phage CC31. When phage cocktail was used against multiple bacterial mixtures, there was a reduction in bacterial load from 10^6^ to 10^3^ CFU/mL within 2 h. All the three characterized phages were found to have a broad host range activity and the prepared phage cocktails were effective against mixed bacterial population that are resistant to meropenem and colistin, two last resort antibiotics. Infections caused by drug resistant bacteria will be a serious threat in the future and the use of virulent bacteriophages in therapy may offer an effective solution.

## Introduction

Bacteriophages are the viruses of bacteria that live in the same ecological niche, where their host bacteria are present (Rohwer, 2003). Phages are generally very specific (species-specific and strain-specific) to their bacterial host but some phages are polyvalent, and can infect more than one species or strain of bacteria (Chibani-Chennoufi et al., 2004). Phage therapy largely involves the treatment of bacterial infections using bacteriophages/phages (Levin and Bull, 2004). Phages with broad host range are mostly chosen for therapy, because of their broad spectrum host-range activity against multiple bacteria. The phages belonging to the order *Caudovirales* (Family-*Myoviridae*, *Siphoviridae*, and *Podoviridae*) with proteinaceous tail, that follow only lytic pathway, are preferred for therapy (Gill and Hyman, 2010). The use of bacteriophages for therapeutic purpose is an old concept that is re-emerging after about a century (Sulakvelidze et al., 2001). Antibiotic resistance has become a human health concern globally as the infections caused by resistant bacteria are becoming difficult to cure (World Health Organization [WHO], 2014; Ventola, 2015). Phage therapy can be one of the alternatives for combating antibiotic resistant bacterial infections (Rios et al., 2016).

*Escherichia coli*, *Klebsiella pneumoniae*, and *Enterobacter cloacae* are Gram-negative bacteria that belong to the family *Enterobacteriaceae*. All the three are enteric pathogens causing serious opportunistic infections in humans (Morens et al., 2004; Schlossberg, 2015). They cause hospital acquired and community acquired infections such as diarrhea, meningitis, urinary tract infections (UTIs), bacteremia, pneumonia, surgical site infections, and sepsis (Schlossberg, 2015). The increasing reports of resistance to carbapenem and colistin, two last resort drugs, among *Enterobacteriaceae* world over (World Health Organization [WHO], 2014) and more particularly so in the developing countries (Ventola, 2015) is a serious threat to their therapeutic use, which prompts search for alternative treatment options. Studies using bacteriophages as an antibacterial agent have shown promising outcomes in both *in vitro* and *in vivo* studies, and therefore phage therapy is being studied as a candidate to cure bacterial infections (McVay et al., 2007; Hung et al., 2011; Cao et al., 2015; Mirzaei and Nilsson, 2015). Phage cocktails have shown broad spectrum activity against many bacterial strains (Mendes et al., 2014; Yen et al., 2017). The characterization of phages for therapeutic purpose involves isolation of potential lytic phages, multi-step *in vitro* characterization, cocktail preparation and purification, dosing and *in vivo* studies. More than 50 *Escherichia* phages belonging to families *Myoviridae*, *Siphoviridae*, and *Podoviridae* have been reported with complete genome sequences^1^. Genome sequenced phages against *Klebsiella* (≈29) and *Enterobacter* (≈10) have been reported much lesser in number^2^. Here, we report characterization of three lytic bacteriophages that showed promising ability to lyse *E. coli*, *K. pneumoniae*, and *Enterobacter* species, report their host range specificity and also efficacy of phage cocktails made using these three phages in various permutations and combinations, in effectively killing combinations of host bacteria using *in vitro* phage killing assay.

## Materials and Methods

### Isolation of Clinical Bacterial Strains for the Study

This study does not include any human subjects; therefore, ethical approval was not required for this study according to national and institutional guidelines. The clinical isolates of *Escherichia coli*, *Klebsiella pneumoniae*, *Enterobacter cloacae, E. hormaechei*, *E. asburiae*, and *E. aerogenes* used in this study were collected from diagnostic centers in Chennai, Tiruchirappalli, and Madurai located in the state of Tamil Nadu in India, during December 2014- September 2016. All the isolates were preserved in 30% glycerol stocks at -20°C. The clinical samples used for bacterial isolation were urine, sputum, pus, blood, wound swab, and bronchial aspirate. Bacterial identification was performed using VITEK identification system (bioMèrieux, Inc., United States) and 16S rRNA analysis. Universal primers, 27F (5′-AGAGTTTGATCCTGGCTCAG-3′) and 1492R (5′-GGTTACCTTGTTACGACTT-3′), were adopted for gene amplification and sequences of 16S rRNA genes were deposited in GenBank. All the clinical isolates were studied for resistance against meropenem and colistin, two last resort antibiotics, using microbroth dilution method following Clinical and Laboratory Standards Institute [CLSI], 2018. For the study, a total of 150 non-repetitive, Gram-negative bacterial isolates belonging to three genera *Escherichia*, *Klebsiella*, and *Enterobacter* were used. The isolated clinical pathogens included 80 *E. coli* isolates, 44 *Klebsiella pneumoniae* isolates and in the case of *Enterobacter* isolates, there were four different species namely *E. cloacae* (*n* = 15), *E. hormaechei* (*n* = 4), *E. asburiae* (*n* = 4), and *E. aerogenes* (*n* = 3) (Table 1). The results for meropenem and colistin resistance screening of these isolates are presented in Supplementary Table S1.

**Table 1:** Host range infection and efficiency of plating (EOP) of the phages *Escherichia* virus myPSH2311, *Klebsiella* virus myPSH1235, and *Enterobacter* virus myPSH1140.

Bacteria	Spot test (%)	High (EOP ≥ 0.5)	Moderate (EOP > 0.1- < 0.5)	Low (EOP ≤ 0.1)	No activity (EOP < 0.001)	Sum of EOP values
***Escherichia* virus** **myPSH2311 lytic activity against different pathotypes of *E. coli***						
EPEC (*n* = 12)	10 (83%)	5	1	1	3	5.30
EHEC (*n* = 10)	6 (60%)	2	0	1	3	1.91
ETEC (*n* = 8)	4 (50%)	3	0	0	1	3.10
EIEC (*n* = 11)	9 (82%)	5	3	0	1	5.55
EAEC (*n* = 14)	10 (71%)	6	0	2	2	5.86
UPEC (*n* = 21)	17 (81%)	11	0	1	5	10.75
Unknown (*n* = 4)	2 (50%)	2	0	0	0	1.60
Total (*n* = 80)	58 (73%)	34	4	5	15	34.07
***Klebsiella* virus** **myPSH1235 lytic activity against different serotypes of *K. pneumoniae***						
Serotype K1 (*n* = 3)	2 (67%)	1	1	0	0	1.20
Serotype K2 (*n* = 7)	5 (71%)	2	1	1	1	2.85
Serotype K5 (*n* = 9)	6 (67%)	2	0	2	2	2.10
Unknown (*n* = 25)	10 (40%)	5	1	1	3	5.85
Total (*n* = 44)	23 (52%)	10	3	4	6	12.0
***Enterobacter* virus** **myPSH1140 lytic activity against different species of *Enterobacter***						
*E. cloacae* (*n* = 15)	15 (100%)	7	3	1	4	8.50
*E. hormaechei* (*n* = 4)	3 (75%)	2	0	0	1	2.72
*E. asburiae* (*n* = 4)	2 (50%)	2	0	0	0	2.10
*E. aerogenes* (*n* = 3)	2 (67%)	2	0	0	0	1.44


*Values represent the lytic activity of the respective phage against the target bacterium. EPEC (enteropathogenic *E. coli*), EHEC (enterohemorrhagic *E. coli*), ETEC (enterotoxigenic *E. coli*), EIEC (enteroinvasive *E. coli*), EAEC (enteroaggregative *E. coli*), UPEC (uropathogenic *E. coli*). EOP was calculated using double agar overlay method. EOP was deemed as ‘High’ if the test bacterium had at least 50% activity (EOP ≥ 0.5) compared to the host bacterium, ‘Moderate’ if between >10 and <50% (EOP > 0.1**-** < 0.5), anything < 10% (EOP ≤ 0.1) was reported as ‘Low.’*

### Phage Isolation and Enrichment

Bacteriophages were isolated from water samples collected from Ganges River near Varanasi in Northern part of India and sewage water treatment plants (secondary treatment stage) from different locations in Chennai, Bangalore, Tirupathi, Vellore, Karur, and Trichy in Southern part of India. Initially, the isolated bacterial strains (one isolate at a time) were grown in Luria-Bertani broth (Himedia, India) medium and were used as a host for phage isolation. Briefly, to a 5 mL of exponentially grown bacterial culture (optical density at 600 nm = 0.6), 10 mL of phage containing water samples was added and incubated at 37°C for 24 h in shaking incubator to enrich the phages against the host bacterium. This mixture was centrifuged for 15 min at 12,000 ×*g* and the supernatant was filtered in 0.22-μm pore-sized membrane syringe filters for separation of phages from other contaminants. The filtrate was used for double-agar overlay method (Li et al., 2016a). Briefly, to the exponentially grown host bacterial culture (400 μL), the phage filtrate (200 μL) was added and incubated for 15 min. To the mixture, 3 mL of molten soft agar (0.75% agar) was added and over-laid onto prepared LB agar plate (1.5% agar). The plates were incubated at 37°C for 10 h and the appearance of clear plaques indicated the presence of phages against the host bacterium. The phage plaques were picked-up from the plate for further purification and the phage titer was determined. For spot test method, the bacterial (using exponentially grown host bacterial culture) lawn was prepared in LB agar plates and 10–50 μL of phage filtrate was placed as a spot over the target bacterial lawn to evaluate the phage activity. The development of clear spots indicated the phage activity and the time taken for bacterial clearance indicated the lytic activity of phages. The bacterium initially used for phage isolation was deemed as a host bacterium against the phage. Multiplicity of infection (MOI) was calculated using the number of phage particles against the potent host bacteria (PFU/CFU).

### Purification of Lytic Phages

The isolated phage lysates were prepared in high titers using phage multiplication strategy (propagation on host bacterium). Briefly, the phages were multiplied using host bacterium for 24 h (day 1) and centrifuged at 6000 ×*g* for 15 min. The collected supernatant was mixed with exponentially grown host bacterium (day 2) and allowed to multiply. Similar passages were carried out for 5 days and evaluated for phage activity/ titer by spot test and double agar overlay method against *E. coli*, *K. pneumoniae*, and *Enterobacter* species. The obtained high titer phages were precipitated using 10% PEG 6000 (polyethylene glycol) and 1 M NaCl. Briefly, to the phage lysate 10% PEG 6000+1 M NaCl was added, mixed gently (did not vortex) and the mixture was stored at 4°C for 24 h. The precipitated phage particles were centrifuged at 15,000 ×*g* for 45 min and the obtained pellet was resuspended in sterile SM buffer (For 1 L: 5.8 g, NaCl; 50 mL, 1 M Tris-HCl [pH 7.5]; 2 g, MgSO_4_.7H_2_O; 5 mL, 2% gelatin). The extraction was carried out by adding equal volume of chloroform and the aqueous phase was sedimented by centrifugation at 18,000 ×*g* for 80 min. The obtained phage particles were dialyzed against phosphate buffer saline (PBS) for 6 h by changing buffer every 2 h and the purified phage suspension was stored at 4°C for further analysis.

### Electron Microscopic Analysis

The purified phage particles (10^5^ PFU/mL) were negatively stained using phosphotungstic acid, PTA (2% [w/v], pH 7.0). Briefly, 10 μL of phage lysate was added over the copper grid and the liquid was allowed to absorb for 10 min. The remaining liquid was removed using tissue paper and the prepared 2% PTA solution (staining solution) was added. After allowing it to stain for 5 min, the excess stain was removed and the grid was washed twice with sterile water. The negatively stained phage particles in the copper grid were allowed to dry at room temperature for 20–30 min and visualized under Transmission Electron Microscopy (FEI-TECNAI G2-20 TWIN, Bionand, Spain). The phage morphology was determined and head/ tail lengths (10 measurements each) were measured using ImageJ software.

### Host-Range Specificity Determination and Efficiency of Plating (EOP)

The lytic activity of isolated phages was tested against the target bacteria. Accordingly, *Escherichia* phage was tested against 80 *E. coli* isolates belonging to pathotypes: EPEC (enteropathogenic *E. coli*), EHEC (enterohemorrhagic *E. coli*), ETEC (enterotoxigenic *E. coli*), EIEC (enteroinvasive *E. coli*), EAEC (enteroaggregative *E. coli*), and UPEC (uropathogenic *E. coli*). *Klebsiella* phage was tested against 37 *K. pneumoniae* isolates that belonged to serotypes K1, K2, K5 and *Enterobacter* phage was tested against 15 *E. cloacae*, 4 *E. hormaechei*, 4 *E. asburiae*, and 3 *E. aerogenes* isolates. Spot test was carried out to assess the host-range specificity of phages against the test bacteria and the resulting positive isolates were again tested for their plaque forming ability in double agar overlay method for calculating efficiency of plating (EOP) (Mirzaei and Nilsson, 2015). EOP was calculated using the number of virus particles infecting the test bacterium against the same titer of virus particles infecting the host bacterium. Accordingly, all the test bacterial strains were grown overnight (16 h) and the concentration of 10^6^–10^9^ (CFU/mL) was used for double agar overlay method. For the assay, 200 μL of bacterial culture was mixed with 100 μL of phage lysate (MOI = 0.01) and EOP was determined using the formula, plaque forming units (PFUs) on the test bacterium/PFU on the host bacterium, evaluated by double agar overlay method. EOP was classified as ‘High,’ ‘Moderate,’ and ‘Low’ based on the productive infection on the target bacterium. EOP was deemed as ‘High’ only if the phage-bacterium combination against the test bacterium had a productive infection of at least 50% (EOP ≥ 0.5) compared to the host bacterium. EOP between >10 and <50% (EOP > 0.1- < 0.5) was considered ‘Moderate’ and EOP < 10% (EOP ≤ 0.1) was recorded as ‘Low’ (Mirzaei and Nilsson, 2015).

### Characterization: Adsorption Rate, Latency Period, and Burst Size

Exponentially grown bacterial cells were mixed with the respective phages at a MOI of 0.001 and incubated at 37°C. Aliquots of 100 μL were removed after every 4 min interval for 40 min and diluted in 4.4 mL LB broth and 0.5 mL of chloroform was added. After incubating the mixture for 30 min at 37°C, the number of non-adsorbed phages was determined subsequently using double agar overlay method. The adsorption curve was constructed using the ratio of non-adsorbed phage particles at different time intervals to the number of initial phages. One-step growth experiment was performed to determine the latent period and burst size (14). Briefly, the bacterial cells (10^8^ CFU/mL) were infected with the phage particles (MOI of 0.001) and allowed to adsorb (based on the adsorption time determined previously) at 37°C. The mixture was then centrifuged at 12,000 ×*g* for 5 min and the pellet was resuspended in 10 mL of LB broth and the incubation was continued at 37°C. The samples were taken at 5 min intervals for 80 min and titrated against the host bacterium. The latent-period was calculated as the duration between the phage adsorbed until the release of phage virions. The burst size of the phage was calculated using the final number of free phage particles to the initial number of phages.

### Phage Stability Studies

Stability studies were conducted at different pH and temperature. For thermal stability tests, phage lysates (10^8^ PFU/mL) were incubated at 4, 20, 35, 45, 50, 55, 60, 70, and 80°C for 60 min in temperature-controlled water bath and immediately transferred to the ice cold condition (-20°C) which was further tested for phage activity using double agar overlay method. The pH stability studies were performed using SM buffer and pH was adjusted using 1N NaOH and 1N HCl. The phage lysates (10^8^ PFU/mL) were incubated at pH 1–14 for 60 min and the aliquots were removed for stability analysis. The results were expressed as phage viability in terms of percentage of initial viral counts. All the stability studies were tested using *E. coli* ec311, *K. pneumoniae* kp235, and *E. cloacae* el140, and the experiments were repeated in triplicates.

### DNA Isolation, Genome Sequencing, and Analysis

The phage DNA was extracted from purified phage particles using phenol-chloroform (24:1) method and precipitated using ethanol (100%). The purified phage DNA was visualized on 0.8% agarose gels and the PE libraries were prepared using Illumina TruSeq Nano DNA library Prep kit. The prepared libraries were sequenced using Illumina Nextseq 500 system (using 2 bp × 150 bp chemistry) at Eurofins Genomics, Bangalore, India. The sequenced raw data was processed to obtain high quality clean reads using Trimmomaticv 0.35 to remove adapter sequences, ambiguous reads (reads with unknown nucleotides “N” larger than 5%), and low-quality sequences [reads with more than 10% quality threshold (QV) < 20 phred score]. The sequenced high quality reads were *de novo* assembled using CLC Genomics Workbench version 9.5.2. Protein-coding and tRNA genes were identified using the final assembly. The transfer-RNA (tRNA) genes were predicted using tRNAscan-SE 2.0 web server while the protein coding genes (CDS) were predicted using FGENESV web server. Functional annotation of the predicted proteins was performed using the amino acid sequences viaBLASTp program online against a custom database of viral proteins in NCBI. Gene ontology (GO) annotations of the genes were determined by the Blast2GO platform. Distribution of GO terms across the categories – Biological Process, Molecular Function and Cellular Component was obtained through WEGOportal ^3^. The NCBI sequence was downloaded from NCBI^2^ for sequence comparison and the scaffolds were then subjected to reference-based assembly via CONTIGuator2. The final assembly generated by CONTIGuator was validated based on sequence homology to known bacteriophage sequences in NCBI via BlastN.

### Composition and Preparation of Phage Cocktails

Phage cocktails containing different compositions of isolated phages under study were prepared and evaluated for activity against target species. For cocktail preparation, two or three different phages were mixed together in equal proportions to obtain a concentration of 10^6^ PFU/mL. Briefly, cocktail EK1 contained *Escherichia* phage plus *Klebsiella* phage; KL2 contained *Klebsiella* phage plus *Enterobacter* phage; EL3 contained *Escherichia* phage plus *Enterobacter* phage and EKL4 contained *Escherichia* phage plus *Klebsiella* phage plus *Enterobacter* phage. The prepared cocktails were tested for *in vitro* phage-killing assay against respective bacterial strains. The results were compared to the activity of phages in cocktails to the activity of phages alone, and one bacterium from each genus was used for this study. Accordingly, 1 mL of host bacterium (6 × 10^7^ CFU/mL) was diluted in LB broth to yield a final concentration of 6 × 10^6^ CFU/mL. For each study, 100 μL (each phage at the concentration of 10^6^ PFU/mL; MOI of 1.0) of bacteriophage suspension was added and the mixture was incubated at 37°C and the aliquots (100 μL) were removed at 0, 2, 4, 6, and 24 h to calculate the reduction in bacterial count. To test the activity of phage cocktails, bacteria were also used in combination similar to phage combinations (Supplementary Table S2). In the case of control experiments, bacteriophage buffer alone was used with the bacterial inoculum and bacterial growth was determined. All the cocktail studies were tested using *E. coli* ec311, *K. pneumoniae* kp235, and *E. cloacae* el140, and the experiments were repeated in triplicates.

## Results

### Nomenclature and Morphological Characterization of Phages

**FIGURE 1:**
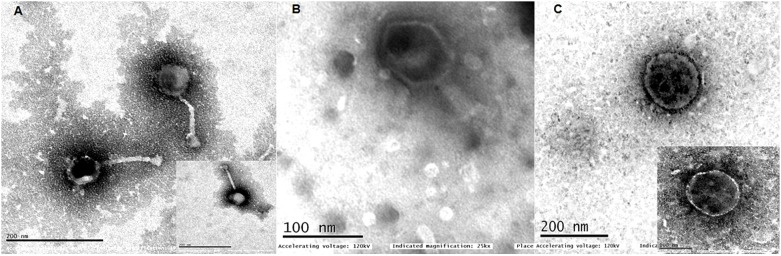
Transmission electron microscopy (TEM) images of **(A)** *Escherichia* virus myPSH2311, **(B)** *Enterobacter* virus myPSH1140, **(C)** *Klebsiella* virus myPSH1235. The smaller images in **(A,C)** represent 100 nm.

The phages were named as *Escherichia* virus myPSH2311, *Klebsiella* virus myPSH1235, and *Enterobacter* virus myPSH1140 following the bacteriophage nomenclature guidelines (Krupovic et al., 2016). Examination of phage morphology by TEM analysis showed that *Escherichia* virus myPSH2311 had an icosahedral head of 33 ± 3.0 nm, a non-contractile tail length of 65 ± 2.5 nm and belonged to genus *Phieco32virus*, *Klebsiella* virus myPSH1235 had the icosahedral head of 80 ± 4.5 nm and very short non-contractile tail that showed the phage belonged to family *Podoviridae* and *Enterobacter* virus myPSH1140 had an elongated head of approximately 80 ± 2.0 nm and long contractile tail of 101 ± 3.5 nm in length indicating that it belonged to the family *Myoviridae* (Figure 1).

### Host-Range Activity Determination and Efficiency of Plating (EOP)

The spot test assay showed that the *Escherichia* virus myPSH2311 had lytic activity against 73% of the tested *E. coli* isolates, *Klebsiella* virus myPSH1235 had activity against 52% *K. pneumoniae* isolates and *Enterobacter* virus myPSH1140 showed activity against 15 *E. cloacae* (*n* = 15), 3 *E. hormaechei* (*n* = 4), 2 *E. asburiae* (*n* = 4), and 2 *E. aerogenes* (*n* = 3) isolates. In the case of double agar overlay method, *Escherichia* virus myPSH2311 had plaques against 43/80 tested *E. coli* isolates, *Klebsiella* virus myPSH1235 had plaques against 17/44 *K. pneumoniae* isolates and *Enterobacter* virus myPSH1140 had plaques against 11/15 *E. cloacae*, 2/4 *E. hormaechei*, 2/4 *E. asburiae*, and 2/3 *E. aerogenes* isolates. The percentage activity difference between the spot test and double agar overlay method was found to be 29.7% for *Escherichia* virus myPSH2311, 30% for *Klebsiella* virus myPSH1235 and for *Enterobacter* virus myPSH1140, it was 30.7% for *E. cloacae*, 40% for *E. hormaechei*, 0% for both *E. asburiae* and *E. aerogenes*. The EOP analysis that was used to differentiate the phage infectivity between spot test and double agar overlay method, showed a different scenario (Table 1). Though, spot test results showed that *Escherichia* virus myPSH1311 produced clear zone (spot) against 58/80 tested *E. coli* isolates; the EOP analysis showed high productive infection in 34/80 *E. coli* isolates whereas against 9 *E. coli* isolates it was moderate or low productive infection and 15/80 *E. coli* isolates had no infection. *Klebsiella* virus myPSH1235 had high productive infection against 10/44 *K. pneumoniae* isolates with 23/44 in spot test assay and the percentage difference was 78.7%. Even if all the EOP results (High + Moderate + Low) were considered for *Klebsiella* phage myPSH1235, the number of isolates producing (17/44) plaques in double agar overlay assay was still lower than the spot test (23/44) results. *Enterobacter* virus myPSH1140 had high productive infection against 7/15 tested *E. cloacae* isolates compared to 15/15 in spot test assay. The same phage showed high productive infection against 2/4 *E. hormaechei*, 2/4 *E. asburiae*, and 2/3 *E. aerogenes*, respectively (Table 1).

### Phage Characterization: One-Step and Stability Studies

**FIGURE 2:**
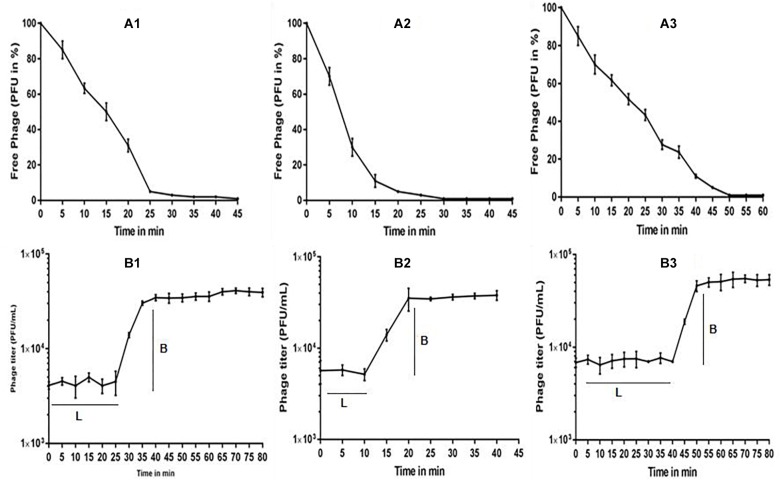
**(A)** Adsorption rate of phages to their bacterial host, **(A1)** *Escherichia* virus myPSH2311, **(A2)** *Enterobacter* virus myPSH1140, **(A3)** *Klebsiella* virus myPSH1235, and **(B)** One-step growth curve experiment of phages, **(B1)** *Escherichia* virus myPSH2311, **(B2)** *Enterobacter* virus myPSH1140, **(B3)** *Klebsiella* virus myPSH1235. Legend within figure B: L-latency period; B-burst size.

**FIGURE 3:**
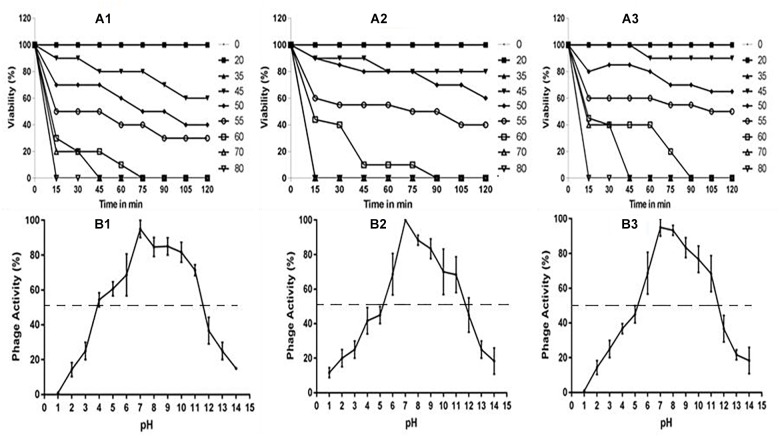
**(A)** Stability of phages at varying temperatures; **(A1)** *Escherichia* virus myPSH2311, **(A2)** *Enterobacter* virus myPSH1140, **(A3)** *Klebsiella* virus myPSH1235 and **(B)** Stability of phages at varying pH at 37°C, **(B1)** *Escherichia* virus myPSH2311, **(B2)** *Enterobacter* virus myPSH1140, **(B3)** *Klebsiella* virus myPSH1235.

The multiplication capacity of phages was determined by one-step growth experiment to analyze the adsorption velocity, latency period and burst size (Figure 2). Accordingly, for *Escherichia* virus myPSH2311, the adsorption velocity was 1.1 × 10^-9^mL/min with latency period of 26 min and the burst size of approximately 110 phages/infected cell. The adsorption velocity for *Klebsiella* virus myPSH1235 was 4.35 × 10^-9^ and the latency period was 40 min with the burst size of 120 phages/infected cell. *Enterobacter* virus myPSH1140 had an adsorption velocity of 2.8 × 10^-9^, a very short latency period of 11 min and a burst size of 135 phages/infected cell (Figure 2). When sensitivity of phages to different pH conditions was determined by exposing them to varying range of pH from 1 to 14 for 60 min, all the three phages were found to be viable from pH 4 up to pH 11, but the phages were inactivated at pH ≤ 3 and ≥ 12 (Figure 3). In the case of thermal stability, all the phages were found to uphold their activity up to 55°C and reduction in activity was observed at higher temperatures (Figure 3). The complete characterization report is available in Supplementary Table S3.

### Genomic Analysis and Annotation

**FIGURE 4:**
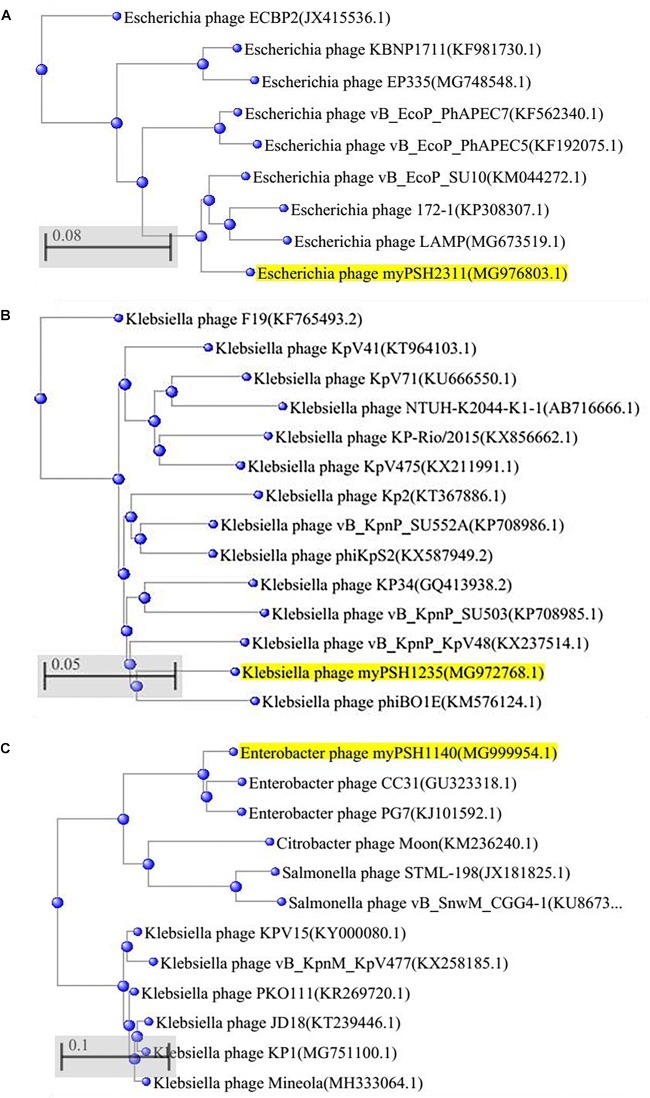
Phylogenetic tree of **(A)** *Escherichia* virus myPSH2311, **(B)** *Klebsiella* virus myPSH1235, and **(C)** *Enterobacter* virus myPSH1140 constructed based on the complete genome sequences of selected bacteriophages in NCBI-BLAST. The tree was produced using BLAST pairwise alignment using Neighbor-Joining method. The query sequence is highlighted in yellow.

The genome of *Escherichia* virus myPSH2311 measured 68,712 bp in size with a GC content of 42.4%. The genome contains 89 proteins or coding sequences (CDS) and it includes 27 proteins of known putative function and 62 hypothetical proteins. A total of 1.01 Gb data was assembled into scaffolds using CLC workbench version 9.5.2, and the assembly size was 5,945,203 bp with the average scaffold size of 12,133 bp. The arranged complete genome of *Escherichia* virus myPSH2311 is closely related to *Escherichia* phage vB_EcoP_SU10 (88%) and *Escherichia* virus phiEco32 (72%) (Figure 4A and Supplementary Figures S1, S2A). The NCBI accession number for this sequence is MG976803. The complete list of all the proteins is available in Supplementary Table S4. *Klebsiella* virus myPSH1235 was found to have a genome size of 45,135 bp with a GC content of 53.7%. The genome contains 49 proteins or CDS, of which 21 were found to have known putative function and 28 were hypothetical proteins. The obtained 1.37 Gb data was assembled into scaffolds using CLC workbench version 9.5.2, and the assembly size was 5,740,807 bp with the average scaffold size of 1,321 bp. The genome was closely related to *Klebsiella* phage vB_KpnP_KpV48 (95%) (Figure 4B and Supplementary Figures S1, S2B). The complete genome of *Klebsiella* virus myPSH1235 is free of toxins or toxin-related genes, and none of the proteins representing a temperate or lysogenic lifestyle was detected (Supplementary Table S4). The NCBI accession number for this phage is MG972768. The complete list of all the proteins is available in Supplementary Table S5. *Enterobacter* virus myPSH1140 was having a genome size of 172,614 bp with a GC content of 39.9%. The gene annotation studies showed 102 proteins with known function and 138 proteins were hypothetical proteins. The obtained 1.22 Gb data was assembled into scaffolds using CLC workbench version 9.5.2, and the assembly size was 5,193,726 bp with the average scaffold size of 6,059 bp. The complete genome was having 92% similarity with *Enterobacter* phage CC31 and 90% similarity with *Enterobacter* phage PG7 (Figure 4C and Supplementary Figures S1, S2C). The NCBI accession number is MG999954. The complete list of all the proteins is available in Supplementary Table S6.

### *In vitro* Activity of Phage Cocktail

**FIGURE 5:**
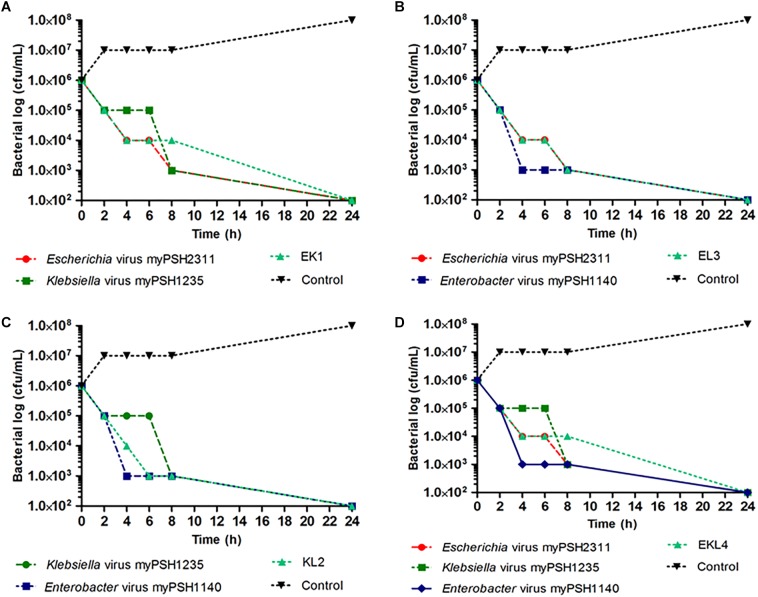
*In vitro* phage activity of prepared phage cocktails. **(A)** activity of *Escherichia* virus myPSH2311 and *Klebsiella* virus myPSH1235 and their combination (EK1); **(B)** activity of *Klebsiella* virus myPSH1235 and *Enterobacter* virus myPSH1140 and their combination (KL2); **(C)** activity of *Escherichia* virus myPSH2311 and *Enterobacter* virus myPSH1140 and their combination (EL3); **(D)** activity of *Escherichia* virus myPSH2311, *Klebsiella* virus myPSH1235, *Enterobacter* virus myPSH1140 and their combination (EKL4) (Control for all the experiments-bacterial growth without antibacterial agents and phages).

Phage cocktails were prepared to evaluate the activity of phages against multiple bacterial strains. When the phage cocktail containing all the three phages was tested against the three meropenem and colistin resistant test bacteria, the growth declined after 2 h from 10^6^ to <10^5^ CFU/mL and at the end of 24 h the bacterial density reached to zero with no viable cells. For EK1 cocktail, >2 fold decrease in bacterial cell count (both *E. coli* and *K. pneumoniae*) was observed after 2 h, for KL2 cocktail, the bacterial cell count (both *K. pneumoniae* and *E. cloacae*) decreased from 10^6^ to 10^3^ CFU/mL within 2 h and for EL3 cocktail, twofold reduction of bacterial cells (both *E. coli* and *E. cloacae*) was observed after 4 h. In phage cocktail containing all the three phages, EKL4, a twofold decrease in bacterial count (*E. coli*, *K. pneumoniae*, *E. cloacae*) was observed in 2 h as comparable to the phages alone. In the case of prepared phage cocktails, all the combinations had similar to better activity in comparison to the phages alone (Figure 5). Our experiment proved the activity of prepared phage cocktails against multiple bacterial genera and showed promising results against meropenem and colistin resistant bacteria *in vitro*.

## Discussion

Phage therapy is currently gaining attention in clinical medicine, because the infections caused by ‘superbugs – multi-drug resistant (MDR) bacteria’ are almost impossible to treat, using available antibiotics (Chan et al., 2013). From clinical point-of-view, bacteriophages are likely to be used in treatment, only when the patient is infected with multi-drug resistant bacteria or during the antibiotic crisis of non-curable infections (Lin et al., 2017). The increasing studies on bacteriophages and phage therapy could put forth phage therapy as one of the alternative treatment options to treat ‘superbug’ infections (Rios et al., 2016). This study is one such effort to characterize bacteriophages infecting Gram-negative bacteria, *E. coli*, *K. pneumoniae*, and *Enterobacter* species.

In the present study, we characterized the phenotypic, morphological, and genomic properties of three bacteriophages that are independently specific in their activity against pathogenic *E. coli, K. pneumoniae*, and *Enterobacter* species. All the three characterized phages belong to order *Caudovirales* that are lytic phages which are generally considered for therapy (*Podoviridae* and *Myoviridae*). There are earlier studies on *Escherichia* phages that showed many of the studied phages are specific to a strain of *E. coli* (e.g., *Escherichia* phage OSYSP infecting only *E. coli* O157:H7) or had a narrow host range activity (Carter et al., 2012; Dalmasso et al., 2016; Baig et al., 2017; Lin et al., 2017). In our study, the characterized *Escherichia* virus myPSH2311 was found to have broad host range activity infecting *E. coli* isolates that belonged to six different pathotypes EPEC, EHEC, ETEC, EIEC, EAEC, and UPEC besides some unknown pathotypes. This is one of the rarest reports where an *Escherichia* phage infecting six different pathotypes of *E. coli* was characterized. But another study also report the broad host range activity of *Escherichia* phage against multiple pathotypes of *E. coli* (Manohar et al., 2018b). The studied *Klebsiella* virus myPSH1235 showed broad host range activity against *K. pneumoniae* belonging to serotypes K1, K2, K5 besides some unknown serotypes. An earlier study showed that a multi-host *Klebsiella* phage ϕK64-1 was capable to infect *Klebsiella* capsular types K1, KN4, KN5, K11, K21, K25, K30, K35, K64, and K69. It is stated that the multi-host infectivity of the bacteriophages is due to the presence of multiple depolymerases in the tail-fibers (Pan et al., 2017). The role of polyvalent phages in phage therapy needs more detailed investigation. The studies on *Enterobacter* phage thus far are very minimal or reports of established *Enterobacter* phages capable of infecting various strains or species are scarce (Li et al., 2016a; Pereira et al., 2016). The characterized *Enterobacter* virus myPSH1140 in our study belonging to *Myoviridae* family showed activity against four species of *Enterobacter* *E. cloacae*, *E. hormaechei*, *E. asburiae*, and *E. aerogenes.* Our study and those of some others strongly suggest that there are bacteriophages that can have broad host range activity against different strains or species or genera of bacteria (Hamdi et al., 2017). A recent study showed that bacteriophages SH6 and SH7 were capable of infecting bacteria from different genera – *E. coli* O157:H7, *Shigella flexneri*, *Salmonella paratyphi*, and *Shigella dysenteriae* (Hamdi et al., 2017). There are studies that showed ‘narrow’ host range specificity of bacteriophages mainly because of the use of standard isolation procedure where single host strain of bacteria is used (Li et al., 2016b; Ross et al., 2016; Hamdi et al., 2017). Future studies should focus on testing the host range activity of bacteriophages but it is also believed that host range activity is not a fixed property of bacteriophages and can evolve overtime (Ross et al., 2016; Hamdi et al., 2017). It should also be noted that we found dissimilarities in the results obtained between spot test and plaque assay in host range activity determination, therefore plaque assay is recommended in order to obtain productive infection for the determination of host range activity of phages (Mirzaei and Nilsson, 2015).

The three characterized bacteriophages had different growth profile, which is one of the important characteristics of lytic bacteriophages. In this study, *Escherichia* virus myPSH2311 (*Podovirus*) had a growth profile with the latent period of 26 min and burst size of 110 phages/infected cell, which is beneficial for therapy. Earlier studies using *Podoviridae* phages showed latency period of ≈15–25 min and burst size of ≈50–80 phages/infected cell (Dalmasso et al., 2016). An earlier study on *Podoviridae* phages infecting *Klebsiella* species was found to have a growth profile with the latency period of 15 min and burst size of ≈50–60 phages/infected cell (Chaturongakul and Ounjai, 2014). In our study, *Klebsiella* virus myPSH1235 belonging to *Podoviridae* family was found to have a latent period of 40 min and burst size of 120 phages/infected cell, which is beneficial for therapy. It was very clear from this study that the growth profile of bacteriophages is not completely dependent on either the family or the host these bacteriophages infect. The bacteriophages belonging to *Myoviridae* family are known to have shorter life cycles and similarly the characterized *Myoviridae* phage (*Enterobacter* phage myPSH1140) had a latent period of 11 min and burst size of 135 phages/infected cell. It was also noted in this and earlier studies that the number of progeny phage particles (burst size) largely depends on the availability of host bacterial cells (Dalmasso et al., 2016). Life cycle parameters of bacteriophages will play significant role in determining both *in vitro* and *in vivo* phage activities (during therapy), because phage multiplication is directly proportional to reduction in bacteria.

The genomes of all the three characterized phages showed 70–100% similarities to the already existing phage genomes in the database. *Escherichia* virus myPSH2311 (68,712 bp) was found to have 88% sequence similarity with *Escherichia* phage vB_EcoP_SU10 (77,327 bp). The endolysin gene (ORF 12) was found to have 98% sequence similarity with *Escherichia* virus phiEco32 and 97% sequence similarity with *Escherichia* phage vB_EcoP_SU10. The DNA injection protein (ORF 17) was detected to have 98% sequence similarity with *Escherichia* phage vB_EcoP_SU10. The genome does not have toxins or toxin-related genes, and none of the genomic markers representing a temperate or lysogenic lifestyle was detected (Supplementary Table S4). *Escherichia* phage vB_EcoP_SU10 is a C3 morphotype phage but *Escherichia* virus myPSH2311 does not have any elongated regions in its morphological characterization (Mirzaei et al., 2014) and related to T4-phages (Petrov et al., 2010). *Klebsiella* virus myPSH1235 was found to have 80% similarity with *Klebsiella* phage vB_KpnP_KpV48. *Klebsiella* virus myPSH1235 has a genome size of 45,135 bp with 49 CDS compared to *Klebsiella* phage vB_KpnP_KpV48, which has a genome size of 44,623 bp with 57 CDS. According to the genome morphology, phylogenetic analysis and sequence similarities, the *Klebsiella* virus myPSH1235 is classified within the genus *Kp34virus*, subfamily *Autographivirinae*, family *Podoviridae*. *Klebsiella* phage vB_KpnP_KpV48 has no capsular specificity and similarly, *Klebsiella* virus myPSH1235 was also found to infect *K. pneumoniae* of capsular types K1, K2 and K5 (Solovieva et al., 2018). From clinical point of view, the *K. pneumoniae* capsular types K1 and K2 are the most virulent strains and that the infections caused by these strains can be eliminated using *Klebsiella* virus myPSH1235 is a promising result, which needs further exploration. *Enterobacter* virus myPSH1140 was found to have a large genome size of 172,614 bp with 240 CDS with 92% similarity with *Enterobacter* phage CC31 (165,540 bp and 279 CDS) and 90% similarity to *Enterobacter* phage PG7 (173,276 and 294 bp). The genome contains 240 proteins or CDS and all the identified proteins were found to have 90–100% sequence similarity with the *Enterobacter* phage CC31 and *Enterobacter* phage PG7. Similarities in the phage genome showed that there is abundance of these phages in the environment and therefore they may be isolated for therapeutic purpose.

Earlier studies on phage cocktails showed promising results in reducing the bacterial load in both *in vitro* and *in vivo* models (Lin et al., 2017; Pan et al., 2017). In our *in vitro* experiments, a phage cocktail that was prepared using the three newly isolated bacteriophages infecting three different genera of bacteria (*Escherichia* virus myPSH2311 against *E. coli*, *Klebsiella* virus myPSH1235 against *K. pneumoniae* and *Enterobacter* virus myPSH1140 against *Enterobacter* species) showed promising results in totally suppressing the bacterial load within 24 h. The phage cocktails reported in this study consisting of multiple phage types could thus prove to be effective as clinical therapeutic agents (Chan et al., 2013; Kęsik-Szeloch et al., 2013). Ours is one of the rarest studies to use phage cocktails against different genera of bacteria. *In vivo* activity of these phage cocktails also showed promising results and the results are published elsewhere (Manohar et al., 2018a).

The strength of our study is that we established the lytic effectiveness of the phages and their cocktail against clinical bacterial isolates of three genera that are known to be pathogenic. Further we also demonstrated the lytic effectiveness of these phages and their cocktail against pathogenic bacteria that were resistant to two last resort antibiotics, meropenem, and colistin. Thus further experiments can be taken up for *in vivo* studies in search of a therapeutic treatment against bacterial infections resistant to these drugs.

## Conclusion

The emergence of multi-drug resistant bacterial strains is a global threat which needs to be addressed by using alternative therapies, such as phage therapy. The three highlighted bacteriophages (*Escherichia* virus myPSH2311 against *E. coli*, *Klebsiella* virus myPSH1235 against *K. pneumoniae* and *Enterobacter* virus myPSH1140 against *Enterobacter* species) and their cocktails showed promising *in vitro* results that may be extended for use as biocontrol agents in some clinical applications. The development of phage cocktails could be considered for the effective treatment of bacterial infections consisting of broad targets. Hopefully, in future, availability of increased repertoire of phages may allow the development of multi-phage cocktail therapy possible.

## Data Availability

All the data that support the findings of this study have been deposited in GenBank with the accession numbers MG976803 (*Escherichia* phage myPSH2311), MG972768 (*Klebsiella* phage myPSH1235), and MG999954 (*Enterobacter* phage myPSH1140).

## Author Contributions

PM and RN collected the isolates and did bacteriophage experiments, undertook the laboratory work, and wrote the initial manuscript. AT, CL, and RN interpreted the data. CL and AT revised and edited the manuscript. All authors approved the final version of the manuscript.

## Conflict of Interest Statement

The authors declare that the research was conducted in the absence of any commercial or financial relationships that could be construed as a potential conflict of interest.

## Footnotes

1 https://www.ncbi.nlm.nih.gov/genome/?term=escherichia+phage

2 https://www.ncbi.nlm.nih.gov/

3 http://wego.genomics.org.cn
