# Supplementary material for: Therapeutic Characterization and Efficacy of Bacteriophage Cocktails Infecting Escherichia coli, Klebsiella pneumoniae, and Enterobacter Species
Source: Front Microbiol. 2019 Mar 21;10:574. doi: 10.3389/fmicb.2019.00574 (PMC6437105; doi:10.3389/fmicb.2019.00574)

**Supplementary information**

**Tables**

**Table S1: Antibiotic resistance pattern of studied *Escherichia*, *Klebsiella* and *Enterobacter* against meropenem and colistin.**

| **Organism/ Antibiotic** | **Meropenem** | **Colistin** | **Both** |
| --- | --- | --- | --- |
|  | **% of resistant isolates** | **% of resistant isolates** | **% resistant to both meropenem and colistin** |
|  |  |  |  |
| ***Escherichia coli* (n=80)** | 79 (n=63) | 40 (n=32) | 38 (n=30) |
| ***Klebsiella pneumoniae* (n=44)** | 70  (n=31) | 43 (n=19) | 41 (n=18) |
| ***Enterobacter cloacae* (n=15)** | 60 (n=09) | 47 (n=07) | 40 (n=06) |
| ***Enterobacter hormaechei*(n=4)** | 75 (n=03) | 25 (n=01) | 25 (n=01) |
| ***Enterobacter asburiae* (n=4)** | 100 (n=04) | 50 (n=02) | 25 (n=01) |
| ***Enterobacter aerogenes* (n=3)** | 33 (n=01) | 33 (n=01) | 33 (n=01) |

**Table S2: Preparation of multiple bacterial cultures and phage combinations to test the activity of phage cocktails.**

| **S.no.** | **Bacterial combination** | **Phage cocktail** |
| --- | --- | --- |
| 1. | *E. coli* and *K. pneumoniae* | **EK1** – *Escherichia* virus myPSH2311 and *Klebsiella* virus myPSH1235 |
| 2. | *K. pneumoniae* and *E. cloacae* | **KL2** - *Klebsiella* virus myPSH1235 and *Enterobacter* virus myPSH1140 |
| 3. | *E. coli* and *E. cloacae* | **EL3** - *Escherichia* virus myPSH2311 and *Enterobacter* virus myPSH1140 |
| 4. | *E. coli, K. pneumoniae* and *E. cloacae* | **EKL4** - *Escherichia* virus myPSH2311, *Klebsiella* virus myPSH1235 and *Enterobacter* virus myPSH1140 |

**Table S3: Taxonomy and morphological characteristics of the three bacteriophages.**

| **Bacteriophage** | ***Escherichia* virus** **myPSH2311** | ***Klebsiella* virus** **myPSH1235** | ***Enterobacter* virus** **myPSH1140** |
| --- | --- | --- | --- |
| **Family** | *Phieco32likevirus* | *Podoviridae* | *Myoviridae* |
| **Capsid size**, nm | 33±3.0 | 80±4.5 | 80±2.0 |
| **Tail length**, nm | 65±2.5 | 30±1.5 | 101±3.5 |
| **Adsorption velocity**, mL/min | 1.1x10^-9^ | 4.35x10^-9^ | 2.8x10^-9^ |
| **Latency period**, min | 26 | 40 | 11 |
| **Burst size**, phage particles/infected cell | 110 | 120 | 135 |

**Table S4: Complete list of proteins/ CDS identified in *Escherichia* virus** **myPSH2311 genome.**

| **ORFs** | **Nucleotide Position** | **Strand** | **Function** |
| --- | --- | --- | --- |
| ORF1 | 466-720 | **+** | Hypothetical protein |
| ORF2 | 713-1345 | **+** | Hypothetical protein |
| ORF3 | 1402-1875 | **+** | Hypothetical protein |
| ORF4 | 2524-3504 | **+** | Terminase large subunit |
| ORF5 | 5341-5745 | **+** | Portal protein |
| ORF6 | 5755-5991 | **+** | Hypothetical protein |
| ORF7 | 5991-7076 | **+** | Scaffolding protein |
| ORF8 | 7667-8065 | **+** | Major head protein |
| ORF9 | 8395-8589 | **+** | Ig-like domain protein |
| ORF10 | 8693-9445 | **+** | Tail tubular protein A |
| ORF11 | 12672-12890 | **+** | Putative holin |
| ORF12 | 12920-13411 | **+** | Lysin |
| ORF13 | 15992-17194 | **+** | Putative receptor protein |
| ORF14 | 18215-19246 | **+** | Hypothetical protein |
| ORF15 | 19259-20041 | **+** | Internal virion D protein |
| ORF16 | 20061-21113 | **+** | Hypothetical protein |
| ORF17 | 21126-22094 | **+** | Putative DNA injection protein |
| ORF18 | 22507-23757 | **+** | Hypothetical protein |
| ORF19 | 28050-28247 | **+** | Hypothetical protein |
| ORF20 | 28516-28355 | **-** | Hypothetical protein |
| ORF21 | 28752-28525 | **-** | Hypothetical protein |
| ORF22 | 29256-29065 | **-** | Hypothetical protein |
| ORF23 | 29413-29270 | **-** | 5’-3’ exonuclease |
| ORF24 | 30934-30041 | **-** | ATP-binding protein |
| ORF25 | 31089-30931 | **-** | GTP-binding protein |
| ORF26 | 31808-31278 | **-** | RNA polymerase ECF sigma factor |
| ORF27 | 32417-32067 | **-** | Hypothetical protein |
| ORF28 | 33221-32475 | **-** | Hypothetical protein |
| ORF29 | 33790-33683 | **-** | Hypothetical protein |
| ORF30 | 33948-33784 | **-** | Hypothetical protein |
| ORF31 | 34417-33914 | **-** | Serine/threonine phosphatase |
| ORF32 | 35323-35156 | **-** | Hypothetical protein |
| ORF33 | 35712-35321 | **-** | Hypothetical protein |
| ORF34 | 35935-35759 | **-** | Hypothetical protein |
| ORF35 | 36112-35945 | **-** | Hypothetical protein |
| ORF36 | 36231-36112 | **-** | Hypothetical protein |
| ORF37 | 36487-36296 | **-** | Hypothetical protein |
| ORF38 | 38304-36496 | **-** | DNA polymerase |
| ORF39 | 38939-38781 | **-** | Hypothetical protein |
| ORF40 | 38939-38781 | **-** | Hypothetical protein |
| ORF41 | 39227-38943 | **-** | Hypothetical protein |
| ORF42 | 39483-39229 | **-** | Hypothetical protein |
| ORF43 | 39762-39553 | **-** | Hypothetical protein |
| ORF44 | 40171-39857 | **-** | Hypothetical protein |
| ORF45 | 41022-40837 | **-** | Hypothetical protein |
| ORF46 | 41338-41009 | **-** | NAD-dependent DNA ligase |
| ORF47 | 41734-41501 | **-** | Hypothetical protein |
| ORF48 | 42026-41751 | **-** | Thymidylate synthase |
| ORF49 | 43361-43182 | **-** | Hypothetical protein |
| ORF50 | 43986-43423 | **-** | DNA-binding protein |
| ORF51 | 44447-44238 | **-** | Hypothetical protein |
| ORF52 | 44644-44447 | **-** | Hypothetical protein |
| ORF53 | 44847-44641 | **-** | Hypothetical protein |
| ORF54 | 45381-44848 | **-** | dCTPdeaminase |
| ORF55 | 45960-45601 | **-** | Hypothetical protein |
| ORF56 | 47229-46894 | **-** | DNA polymerase |
| ORF57 | 48785-47445 | **-** | Primase/helicase activity |
| ORF58 | 49303-49151 | **-** | Hypothetical protein |
| ORF59 | 50328-50191 | **-** | Hypothetical protein |
| ORF60 | 50615-50373 | **-** | Hypothetical protein |
| ORF61 | 50876-50628 | **-** | ATP-grasp enzyme |
| ORF62 | 51730-51593 | **-** | Hypothetical protein |
| ORF63 | 52017-51773 | **-** | Hypothetical protein |
| ORF64 | 52278-52030 | **-** | ATP-grasp enzyme |
| ORF65 | 53096-52959 | **-** | Hypothetical protein |
| ORF66 | 53389-53141 | **-** | Hypothetical protein |
| ORF67 | 54490-53395 | **-** | ATP-grasp enzyme |
| ORF68 | 56029-55058 | **-** | Hypothetical protein |
| ORF69 | 57239-56040 | **-** | Hypothetical protein |
| ORF70 | 57613-57251 | **-** | Hypothetical protein |
| ORF71 | 57768-57616 | **-** | Hypothetical protein |
| ORF72 | 58459-57995 | **-** | Hypothetical protein |
| ORF73 | 58692-58459 | **-** | Hypothetical protein |
| ORF74 | 59498-59250 | **-** | Hypothetical protein |
| ORF75 | 59730-59500 | **-** | Hypothetical protein |
| ORF76 | 60050-59739 | **-** | Transcriptional regulator |
| ORF77 | 60536-60405 | **-** | Hypothetical protein |
| ORF78 | 61531-61313 | **-** | Hypothetical protein |
| ORF79 | 61789-61532 | **-** | Hypothetical protein |
| ORF80 | 62561-62265 | **-** | Hypothetical protein |
| ORF81 | 63150-62761 | **-** | Hypothetical protein |
| ORF82 | 63540-63367 | **-** | Hypothetical protein |
| ORF83 | 63959-63537 | **-** | Hypothetical protein |
| ORF84 | 64615-64397 | **-** | Hypothetical protein |
| ORF85 | 65284-64727 | **-** | Hypothetical protein |
| ORF86 | 65945-65456 | **-** | Hypothetical protein |
| ORF87 | 68097-67732 | **-** | Hypothetical protein |
| ORF88 | 68551-68291 | **-** | Hypothetical protein |
| ORF89 | 68690-68541 | **-** | Hypothetical protein |

**Table S5: Complete list of proteins/ CDS identified in *Klebsiella* virus** **myPSH1235 genome.**

| **ORFs** | **Nucleotide Position** | **Strand** | **Function** |
| --- | --- | --- | --- |
| ORF1 | 118-903 | **+** | DNA primase |
| ORF2 | 900-1094 | **+** | Hypothetical protein |
| ORF3 | 1260-2375 | **+** | Putative DNA primase |
| ORF4 | 2606-2761 | **+** | Hypothetical protein |
| ORF5 | 2758-2940 | **+** | Hypothetical protein |
| ORF6 | 3291-5306 | **+** | DNA polymerase I |
| ORF7 | 5303-5524 | **+** | Hypothetical protein |
| ORF8 | 5765-6079 | **+** | Hypothetical protein |
| ORF9 | 6262-7077 | **+** | Putative phosphoesterase |
| ORF10 | 7097-7285 | **+** | Hypothetical protein |
| ORF11 | 7341-8183 | **+** | Large tegument protein |
| ORF12 | 8237-8491 | **+** | Hypothetical protein |
| ORF13 | 8492-8770 | **+** | Hypothetical protein |
| ORF14 | 8770-9144 | **+** | Hypothetical protein |
| ORF15 | 9308-9505 | **+** | Hypothetical protein |
| ORF16 | 9508-9666 | **+** | Hypothetical protein |
| ORF17 | 9666-10100 | **+** | Hypothetical protein |
| ORF18 | 10249-11049 | **+** | 5’-3’ exonuclease |
| ORF19 | 11006-11209 | **+** | Hypothetical protein |
| ORF20 | 11200-11622 | **+** | DNA endonuclease VII |
| ORF21 | 11784-12113 | **+** | Polynucleotide kinase/ phosphatase |
| ORF22 | 12110-12424 | **+** | Hypothetical protein |
| ORF23 | 12566-15034 | **+** | RNA polymerase |
| ORF24 | 15058-15498 | **+** | Hypothetical protein |
| ORF25 | 15495-15758 | **+** | Hypothetical protein |
| ORF26 | 16809-17363 | **+** | Head-tail connector |
| ORF27 | 17378-18220 | **+** | Putative scaffolding protein |
| ORF28 | 18246-19265 | **+** | Capsid protein |
| ORF29 | 19546-19725 | **+** | Hypothetical protein |
| ORF30 | 19818-20378 | **+** | Tail tubular protein |
| ORF31 | 20971-23229 | **+** | Tail tubular protein |
| ORF32 | 23231-23818 | **+** | Internal virion protein B |
| ORF33 | 23836-26520 | **+** | Hypothetical protein |
| ORF34 | 26571-30836 | **+** | Internal core protein |
| ORF35 | 31799-32101 | **+** | DNA muturase A |
| ORF36 | 32101-33957 | **+** | DNA muturase B |
| ORF37 | 33957-34331 | **+** | Hypothetical protein |
| ORF38 | 34343-34525 | **+** | Hypothetical protein |
| ORF39 | 34525-34929 | **+** | Putative spanin protein |
| ORF40 | 34922-35173 | **+** | Putative holin |
| ORF41 | 39073-39288 | **+** | Hypothetical protein |
| ORF42 | 39448-40023 | **+** | Hypothetical protein |
| ORF43 | 40089-40331 | **+** | Hypothetical protein |
| ORF44 | 40393-40614 | **+** | Hypothetical protein |
| ORF45 | 40607-40870 | **+** | Hypothetical protein |
| ORF46 | 40879-41058 | **+** | Hypothetical protein |
| ORF47 | 41055-41261 | **+** | Hypothetical protein |
| ORF48 | 41643-43316 | **+** | Hypothetical protein |
| ORF49 | 43316-44362 | **+** | Putative peptidase |

**Table S6: Complete list of proteins/ CDS identified in *Enterobacter* virus** **myPSH1140 genome.**

| **ORFs** | **Nucleotide Position** | **Strand** | **Function** |
| --- | --- | --- | --- |
| ORF1 | 832-515 | **-** | Hypothetical protein |
| ORF2 | 1341-883 | **-** | Protector from prophage-induced early lysis |
| ORF3 | 4018-1805 | **-** | Protector from prophage-induced early lysis |
| ORF4 | 4230-4024 | **-** | Hypothetical protein |
| ORF5 | 4508-4224 | **-** | Hypothetical protein |
| ORF6 | 4709-4554 | **-** | Hypothetical protein |
| ORF7 | 6590-4749 | **-** | DNA topoisomerase |
| ORF8 | 7025-6639 | **-** | Hypothetical protein |
| ORF9 | 8639-7104 | **-** | Hypothetical protein |
| ORF10 | 8932-8672 | **-** | Hypothetical protein |
| ORF11 | 9200-9024 | **-** | Hypothetical protein |
| ORF12 | 9522-9187 | **-** | Hypothetical protein |
| ORF13 | 10031-9522 | **-** | Hypothetical protein |
| ORF14 | 10928-10491 | **-** | Hypothetical protein |
| ORF15 | 11672-10983 | **-** | Exonuclease A |
| ORF16 | 11911-11669 | **-** | Hypothetical protein |
| ORF17 | 13230-13257 | **-** | DNA helicase |
| ORF18 | 13571-13257 | **-** | Hypothetical protein |
| ORF19 | 14323-13571 | **-** | Anti-sigma factor |
| ORF20 | 15086-14409 | **-** | ADP-ribosylase |
| ORF21 | 15338-15147 | **-** | Hypothetical protein |
| ORF22 | 15757-15353 | **-** | Hypothetical protein |
| ORF23 | 16079-15855 | **-** | Hypothetical protein |
| ORF24 | 16429-16178 | **-** | Hypothetical protein |
| ORF25 | 17489-16971 | **-** | dCTPase, dUTPase, dCDPase, dUDPase |
| ORF26 | 17819-17535 | **-** | Hypothetical protein |
| ORF27 | 18841-17816 | **-** | DNA primase subunit |
| ORF28 | 19349-18885 | **-** | Hypothetical protein |
| ORF29 | 19553-19365 | **-** | Hypothetical protein |
| ORF30 | 19876-19550 | **-** | Hypothetical protein |
| ORF31 | 20064-19873 | **-** | Hypothetical protein |
| ORF32 | 20342-20061 | **-** | Hypothetical protein |
| ORF33 | 21776-20400 | **-** | DNA primase/ helicase |
| ORF34 | 22221-21853 | **-** | Head vertex assembly chaperone |
| ORF35 | 24651-23575 | **-** | Beta-glucosyltransferase |
| ORF36 | 25478-24648 | **-** | Hypothetical protein |
| ORF37 | 26742-26002 | **-** | dCMPhydroxymethylase |
| ORF38 | 27179-26739 | **-** | Hypothetical protein |
| ORF39 | 29950-27242 | **-** | DNA polymerase |
| ORF40 | 30209-30048 | **-** | Hypothetical protein |
| ORF41 | 30574-30209 | **-** | Translational repressor protein |
| ORF42 | 31139-30576 | **-** | Clamp loader subunit, DNA polymerase |
| ORF43 | 32095-31136 | **-** | Clamp loader subunit, DNA polymerase |
| ORF44 | 32841-32155 | **-** | Sliding clamp, DNA polymerase accessory |
| ORF45 | 33275-32850 | **-** | RNA polymerase binding protein |
| ORF46 | 33476-33285 | **-** | Hypothetical protein |
| ORF47 | 35161-33474 | **-** | Recombination endonuclease subunit |
| ORF48 | 36180-35158 | **-** | Endonuclease subunit |
| ORF49 | 37010-36684 | **-** | Hypothetical protein |
| ORF50 | 37760-37227 | **-** | RNA polymerase sigma factor |
| ORF51 | 38371-38120 | **-** | Hypothetical protein |
| ORF52 | 38595-38368 | **-** | Hypothetical protein |
| ORF53 | 38930-38592 | **-** | Hypothetical protein |
| ORF54 | 40214-39900 | **-** | Glutaredoxin |
| ORF55 | 40464-40189 | **-** | Hypothetical protein |
| ORF56 | 40749-40564 | **-** | Anaerobic NTP reductase, small subunit |
| ORF57 | 42868-41039 | **-** | Anaerobic NTP reductase, small subunit |
| ORF58 | 43338-42865 | **-** | Recombinase endonuclease VII |
| ORF59 | 43831-43373 | **-** | Protease inhibitor |
| ORF60 | 43961-43815 | **-** | Hypothetical protein |
| ORF61 | 44257-43946 | **-** | Hypothetical protein |
| ORF62 | 44430-44257 | **-** | Hypothetical protein |
| ORF63 | 44961-44668 | **-** | Hypothetical protein |
| ORF64 | 45251-44958 | **-** | Hypothetical protein |
| ORF65 | 45583-45248 | **-** | Hypothetical protein |
| ORF66 | 45926-45669 | **-** | Hypothetical protein |
| ORF67 | 47216-47010 | **-** | Hypothetical protein |
| ORF68 | 47641-47204 | **-** | Hypothetical protein |
| ORF69 | 48411-47635 | **-** | Hypothetical protein |
| ORF70 | 48918-48577 | **-** | Hypothetical protein |
| ORF71 | 49358-48942 | **-** | Hypothetical protein |
| ORF72 | 49573-49358 | **-** | Hypothetical protein |
| ORF73 | 50252-49749 | **-** | Hypothetical protein |
| ORF74 | 50613-50263 | **-** | Hypothetical protein |
| ORF75 | 50747-50601 | **-** | Hypothetical protein |
| ORF76 | 51763-50786 | **-** | Hypothetical protein |
| ORF77 | 52097-51834 | **-** | Hypothetical protein |
| ORF78 | 52243-52094 | **-** | Hypothetical protein |
| ORF79 | 52455-52243 | **-** | Hypothetical protein |
| ORF80 | 53828-52818 | **-** | Hypothetical protein |
| ORF81 | 54250-53825 | **-** | Hypothetical protein |
| ORF82 | 54801-54247 | **-** | Hypothetical protein |
| ORF83 | 55463-55011 | **-** | Hypothetical protein |
| ORF84 | 55730-55485 | **-** | Hypothetical protein |
| ORF85 | 56001-55723 | **-** | Hypothetical protein |
| ORF86 | 56191-55985 | **-** | Hypothetical protein |
| ORF87 | 56475-56188 | **-** | Hypothetical protein |
| ORF88 | 56747-56478 | **-** | Hypothetical protein |
| ORF89 | 57312-56854 | **-** | Hypothetical protein |
| ORF90 | 57739-57419 | **-** | Hypothetical protein |
| ORF91 | 58070-57795 | **-** | Lysis inhibition regulator |
| ORF92 | 58290-58078 | **-** | Hypothetical protein |
| ORF93 | 58733-58350 | **-** | Hypothetical protein |
| ORF94 | 59028-58897 | **-** | Hypothetical protein |
| ORF95 | 59640-59056 | **-** | Thymidine kinase |
| ORF96 | 60289-59834 | **-** | Hypothetical protein |
| ORF97 | 61028-60702 | **-** | Valyl-tRNAsynthetase modifier |
| ORF98 | 62059-61595 | **-** | Site-specific RNA endonuclease |
| ORF99 | 62384-62118 | **-** | Hypothetical protein |
| ORF100 | 62494-62348 | **-** | Hypothetical protein |
| ORF101 | 63351-62932 | **-** | Hypothetical protein |
| ORF102 | 63896-63348 | **-** | Hypothetical protein |
| ORF103 | 64586-64173 | **-** | Endonuclease V, N-glycosylase UV repair enzyme |
| ORF104 | 64941-64645 | **-** | Hypothetical protein |
| ORF105 | 65422-64928 | **-** | Lysozyme murein hydrolase |
| ORF106 | 65869-65456 | **-** | Nudix hydrolase |
| ORF107 | 66162-65932 | **-** | Hypothetical protein |
| ORF108 | 66470-66159 | **-** | Hypothetical protein |
| ORF109 | 66829-66467 | **-** | Hypothetical protein |
| ORF110 | 67164-66826 | **-** | Hypothetical protein |
| ORF111 | 67501-67148 | **-** | Hypothetical protein |
| ORF112 | 67878-67504 | **-** | Hypothetical protein |
| ORF113 | 68829-67912 | **-** | Hypothetical protein |
| ORF114 | 69487-69254 | **-** | Hypothetical protein |
| ORF115 | 69780-69496 | **-** | Hypothetical protein |
| ORF116 | 71078-70476 | **-** | Hypothetical protein |
| ORF117 | 71628-71486 | **-** | Hypothetical protein |
| ORF118 | 71911-71651 | **-** | Hypothetical protein |
| ORF119 | 73244-73038 | **-** | Hypothetical protein |
| ORF120 | 73952-73728 | **-** | Hypothetical protein |
| ORF121 | 74405-73956 | **-** | Hypothetical protein |
| ORF122 | 75352-74855 | **-** | Hypothetical protein |
| ORF123 | 75737-75363 | **-** | Hypothetical protein |
| ORF124 | 76080-75856 | **-** | Hypothetical protein |
| ORF125 | 76679-76122 | **-** | Hypothetical protein |
| ORF126 | 78867-78643 | **-** | Hypothetical protein |
| ORF127 | 79607-79428 | **-** | Putative membrane protein |
| ORF128 | 79981-79673 | **-** | Ip7 protein |
| ORF129 | 80436-79978 | **-** | Hypothetical protein |
| ORF130 | 80687-80433 | **-** | Chaperone for tail fiber formation |
| ORF131 | 81412-80684 | **-** | Deoxynucleoside monophosphate kinase |
| ORF132 | 81752-81414 | **-** | Tail completion and sheath stabilizer |
| ORF133 | 82916-82086 | **-** | DNA end protector protein |
| ORF134 | 83365-82916 | **-** | Head completion protein |
| ORF135 | 83415-83990 | **+** | Baseplate hub subunit and tail lysozyme |
| ORF136 | 83990-85726 | **+** | Baseplate hub subunit and tail lysozyme |
| ORF137 | 85734-86237 | **+** | Hypothetical protein |
| ORF138 | 86238-86534 | **+** | Hypothetical protein |
| ORF139 | 86539-88485 | **+** | Baseplate wedge initiator |
| ORF140 | 88482-91580 | **+** | Baseplate wedge initiator |
| ORF141 | 91573-92583 | **+** | Baseplate wedge initiator |
| ORF142 | 92644-93552 | **+** | Baseplate wedge subunit |
| ORF143 | 93549-95357 | **+** | Baseplate wedge subunit and tail pin |
| ORF144 | 95357-96019 | **+** | Baseplate wedge subunit and tail pin |
| ORF145 | 96019-97569 | **+** | Short tail fiber protein |
| ORF146 | 97578-98990 | **+** | Fibritin neck whisker protein |
| ORF147 | 99021-99959 | **+** | Neck protein |
| ORF148 | 99956-100756 | **+** | Neck protein |
| ORF149 | 100847-101632 | **+** | Tail sheath stabilizer and completion |
| ORF150 | 101632-102129 | **+** | Large terminase protein |
| ORF151 | 102113-103945 | **+** | Large terminase protein |
| ORF152 | 103979-105961 | **+** | Tail sheath protein |
| ORF153 | 106743-107234 | **+** | Tail tube protein |
| ORF154 | 107880-109448 | **+** | Portal vertex protein of head |
| ORF155 | 109697-110122 | **+** | Prohead core protein |
| ORF156 | 110122-110763 | **+** | Prohead core protein |
| ORF157 | 110797-111612 | **+** | Prohead core protein |
| ORF158 | 111635-113197 | **+** | Major capsid protein |
| ORF159 | 113283-114566 | **+** | Head vertex protein |
| ORF160 | 116148-115144 | **-** | RNA ligase 2 activity |
| ORF161 | 116405-116157 | **-** | Hypothetical protein |
| ORF162 | 116873-116520 | **-** | Hypothetical protein |
| ORF163 | 118518-117766 | **-** | Inhibitor of prohead protease |
| ORF164 | 118571-120073 | **+** | RNA-DNA and DNA-DNA helicase, ATPase |
| ORF165 | 120081-120320 | **+** | Putative split helicase |
| ORF166 | 120540-120373 | **-** | Hypothetical protein |
| ORF167 | 120791-120564 | **-** | Hypothetical protein |
| ORF168 | 121204-120791 | **-** | Recombination, repair and ssDNA binding protein |
| ORF169 | 121679-121281 | **-** | Baseplate wedge subunit |
| ORF170 | 122308-121679 | **-** | Baseplate hub subunit |
| ORF171 | 122457-123110 | **+** | Baseplate hub subunit |
| ORF172 | 123224-124267 | **+** | Baseplate hub subunit |
| ORF173 | 124314-123751 | **-** | Baseplate distal hub subunit |
| ORF174 | 124748-126511 | **+** | Baseplate hub subunit, tail length |
| ORF175 | 126521-127570 | **+** | Baseplate subunit |
| ORF176 | 127570-128532 | **+** | Baseplate subunit |
| ORF177 | 128851-128562 | **-** | Hypothetical protein |
| ORF178 | 129259-128912 | **-** | RNA polymerase ADP-ribosylase |
| ORF179 | 132330-131278 | **-** | DNA ligase |
| ORF180 | 133024-132794 | **-** | Hypothetical protein |
| ORF181 | 133884-133021 | **-** | Hypothetical protein |
| ORF182 | 134050-133874 | **-** | Hypothetical protein |
| ORF183 | 134401-134198 | **-** | Hypothetical protein |
| ORF184 | 134565-134398 | **-** | Hypothetical protein |
| ORF185 | 135035-134565 | **-** | Hypothetical protein |
| ORF186 | 135436-135074 | **-** | Hypothetical protein |
| ORF187 | 136029-135508 | **-** | Hypothetical protein |
| ORF188 | 136428-136054 | **-** | Hypothetical protein |
| ORF189 | 136640-136497 | **-** | Hypothetical protein |
| ORF190 | 136933-136754 | **-** | Hypothetical protein |
| ORF191 | 137505-137251 | **-** | Lysis inhibition accessory protein, rapid lysis protein |
| ORF192 | 137963-137640 | **-** | Head assembly cochaperone with GroEL |
| ORF193 | 138349-138020 | **-** | Hypothetical protein |
| ORF194 | 138908-138342 | **-** | Deoxycytidylatedeaminase |
| ORF195 | 139252-139472 | **+** | Hypothetical protein |
| ORF196 | 139765-139472 | **-** | Hypothetical protein |
| ORF197 | 139980-139765 | **-** | Hypothetical protein |
| ORF198 | 140188-139997 | **-** | Hypothetical protein |
| ORF199 | 141075-140188 | **-** | Polynucleotide 5’-kinase and 3’-phosphatase |
| ORF200 | 141410-141072 | **-** | Hypothetical protein |
| ORF201 | 141615-141400 | **-** | Hypothetical protein |
| ORF202 | 142091-141612 | **-** | Hypothetical protein |
| ORF203 | 142238-142140 | **-** | Hypothetical protein |
| ORF204 | 142875-142519 | **-** | Putative membrane protein |
| ORF205 | 143369-142963 | **-** | Inhibitor of host transcription |
| ORF206 | 144539-143433 | **-** | RNA ligase 1 and tail fiber attachment catalyst |
| ORF207 | 144973-144560 | **-** | Aerobic NDP reductase small subunit |
| ORF208 | 145575-145000 | **-** | Aerobic NDP reductase small subunit |
| ORF209 | 148517-146262 | **-** | Ribonucleoside-diphosphatereductase subunit |
| ORF210 | 148815-148504 | **-** | Hypothetical protein |
| ORF211 | 149902-149042 | **-** | dTMP (thymidylate) synthase |
| ORF212 | 150249-149902 | **-** | Hypothetical protein |
| ORF213 | 150556-150236 | **-** | Hypothetical protein |
| ORF214 | 151136-150543 | **-** | Dihydrofolatereductase |
| ORF215 | 152025-151699 | **-** | Hypothetical protein |
| ORF216 | 152309-152025 | **-** | Hypothetical protein |
| ORF217 | 154343-153424 | **-** | Single-stranded DNA binding protein |
| ORF218 | 155008-154355 | **-** | Loader of DNA helicase |
| ORF219 | 155187-155005 | **-** | Late promoter transcriptional accessory protein |
| ORF220 | 155557-155282 | **-** | Double-stranded DNA binding protein |
| ORF221 | 156503-155565 | **-** | RnaseH |
| ORF222 | 156558-160352 | **+** | Long tail fiber, proximal subunit |
| ORF223 | 160352-161500 | **+** | Hinge connector of long tail fiber |
| ORF224 | 161560-162213 | **+** | Hinge connector of long tail fiber distal |
| ORF225 | 165757-166413 | **+** | Holing lysis mediator |
| ORF226 | 166694-166422 | **+** | Anti-sigma 70 protein |
| ORF227 | 167019-166762 | **+** | Hypothetical protein |
| ORF228 | 167369-167019 | **-** | Hypothetical protein |
| ORF229 | 167619-167362 | **-** | Hypothetical protein |
| ORF230 | 167749-167627 | **-** | Hypothetical protein |
| ORF231 | 168029-167724 | **-** | Hypothetical protein |
| ORF232 | 168412-168029 | **-** | Hypothetical protein |
| ORF233 | 168819-168409 | **-** | Hypothetical protein |
| ORF234 | 169478-168834 | **-** | Activator of middle period transcription |
| ORF235 | 169860-169588 | **-** | Hypothetical protein |
| ORF236 | 171423-170044 | **-** | DNA topoisomerase II medium subunit |
| ORF237 | 171564-171430 | **-** | Hypothetical protein |
| ORF238 | 172191-171745 | **-** | Nucleoid disruption protein |
| ORF239 | 172454-172248 | **-** | Hypothetical protein |
| ORF240 | 172584-172465 | **-** | Hypothetical protein |

**Figure S1: Similarities in genome arrangement of sequenced bacteriophage genomes;** A) Circos plot depicting sequence similarities of *Escherichia* virus myPSH2311 against *Escherichia* phage vB_EcoP_SU10, B) Circos plot depicting sequence similarities of *Klebsiella* virus myPSH1235 against *Klebsiella* phage vB_KpnP_KpV48, C) Similarity in genome arrangement of *Enterobacter* virus myPSH1140 across *Enterobacter* phage PG7. The red color signifies high sequence similarity followed by orange, green and blue.

**
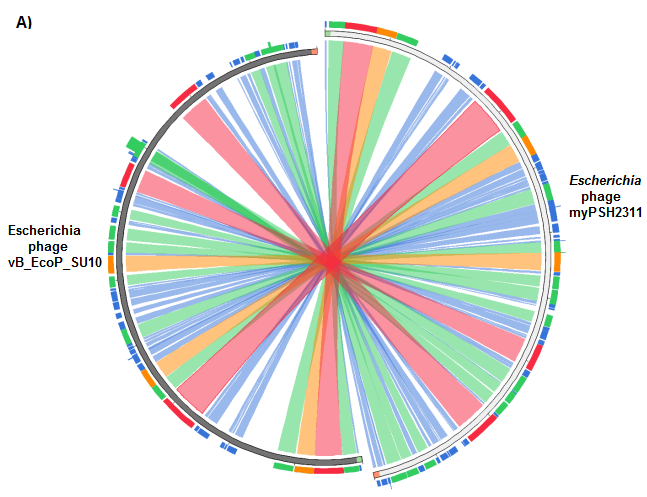
**

**
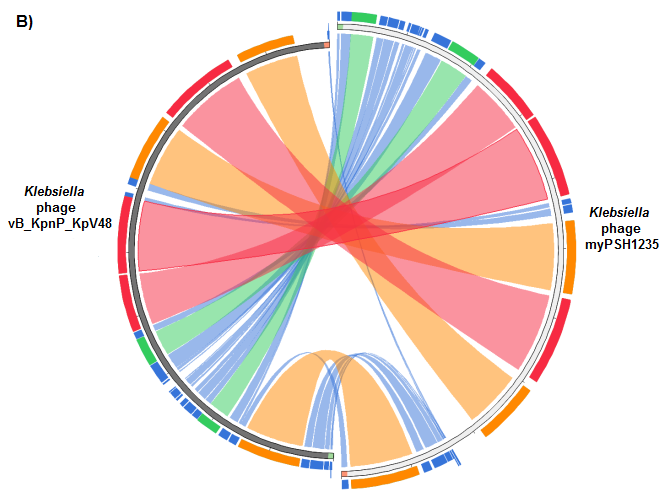
**

**
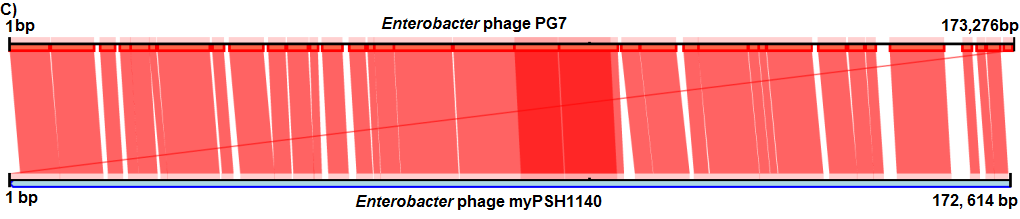
**

**Figure S2: Comparative analysis of whole genome sequences using wgVISTA database.** A) Depicts the sequence similarities of *Escherichia* virus myPSH2311 against *Escherichia* phage vB_EcoP_SU10, B) Depicts the sequence similarities of *Klebsiella* virus myPSH1235 against *Klebsiella* phage vB_KpnP_KpV48, C) Depicts the similarity in genome arrangement of *Enterobacter* virus myPSH1140 across *Enterobacter* phage PG7.


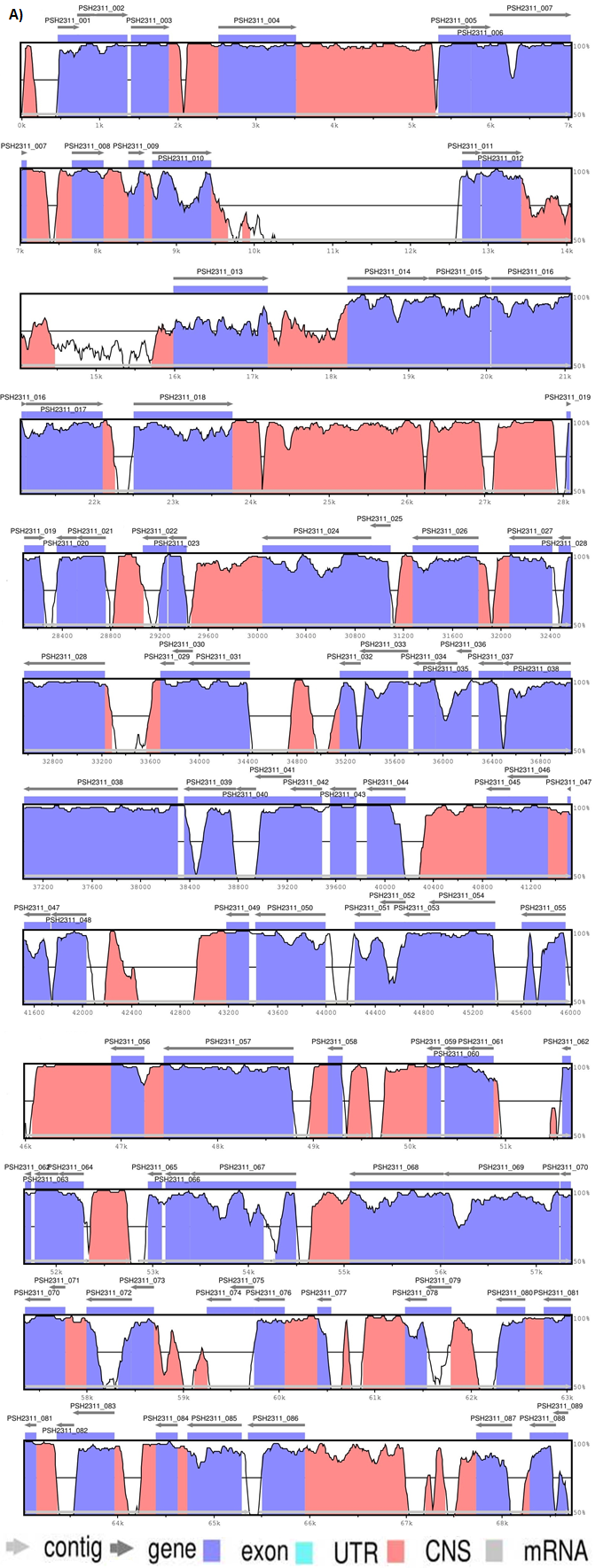


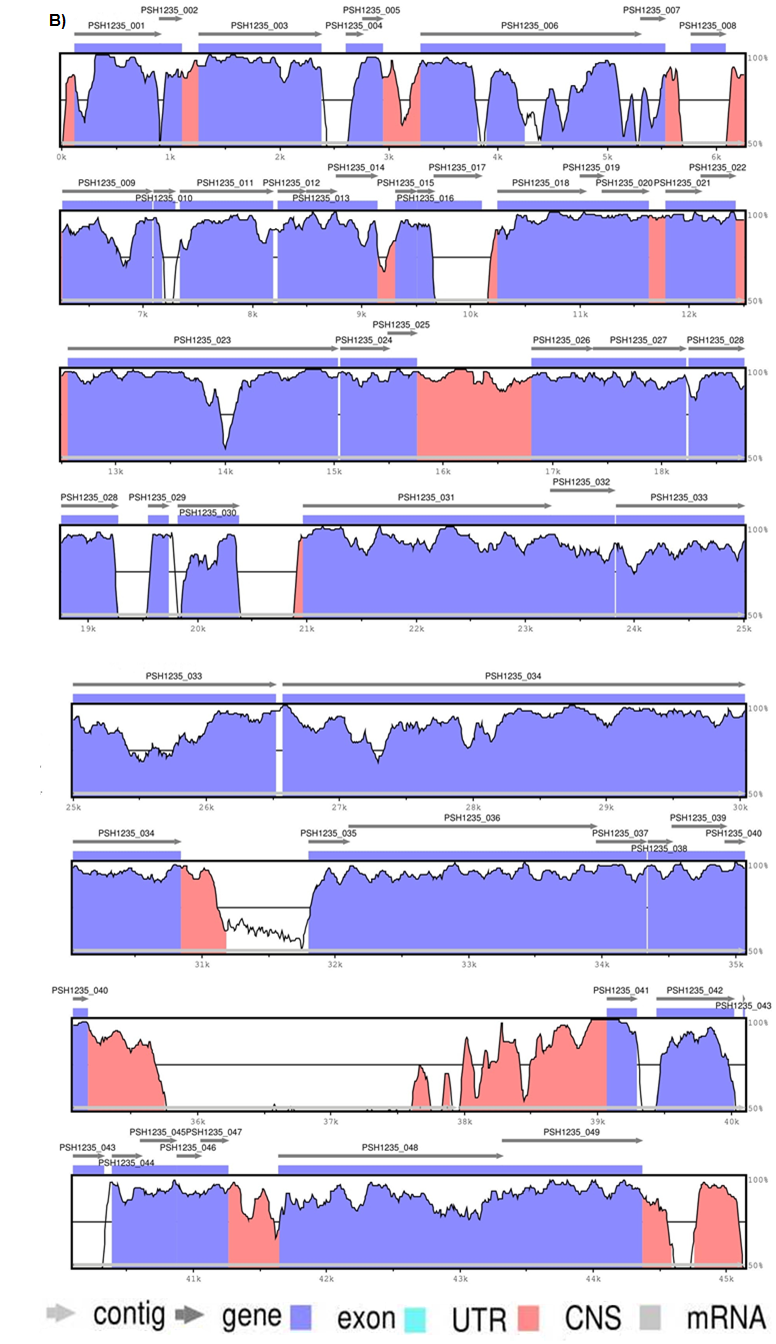


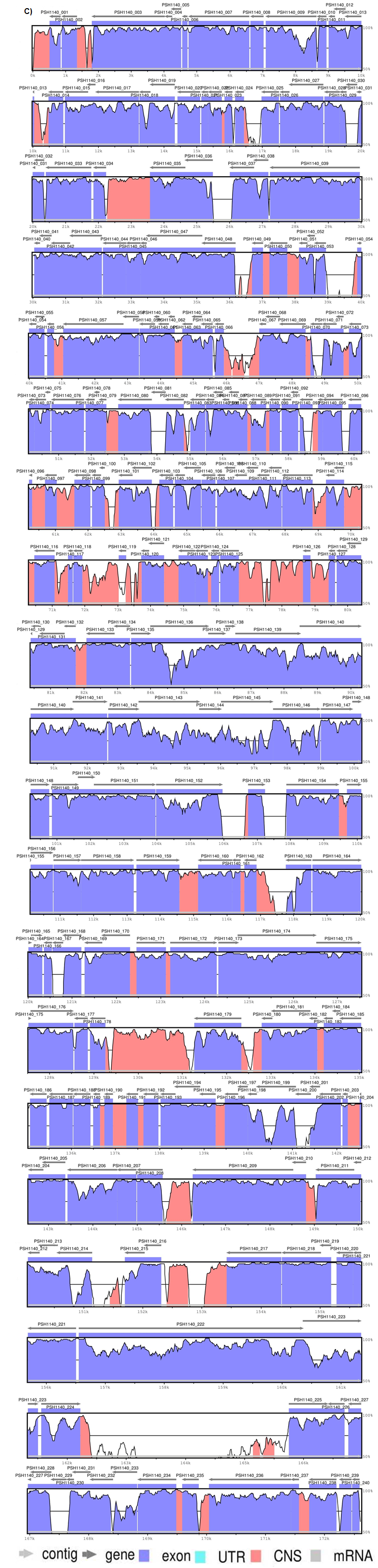

Supplement: Supplementary file 1 [file Data_Sheet_1.docx]
